# Polydnaviruses as Symbionts and Gene Delivery Systems

**DOI:** 10.1371/journal.ppat.1002757

**Published:** 2012-07-05

**Authors:** Michael R. Strand, Gaelen R. Burke

**Affiliations:** Department of Entomology, University of Georgia, Athens, Georgia, United States of America; University of Florida, United States of America

Textbooks define viruses as infectious agents with nucleic acid genomes (RNA or DNA), which replicate inside living host cells to produce particles (virions) that can transfer the genome to other cells [1], [2]. The Polydnaviridae was recognized as a family of viruses in 1995, and is currently divided into two genera named the *Bracovirus* and *Ichnovirus* [3]. Polydnavirus (PDV) virions consist of enveloped nucleocapsids and package multiple circular, double-stranded (ds) DNAs with aggregate sizes that range from 190 to more than 500 kbp [4]. PDVs are also strictly associated with insects called parasitoid wasps (Hymenoptera), which are free living nectar feeders as adults but which develop during their immature stages by feeding inside the body of another insect (the host) [3], [4]. Recent studies, however, indicate that PDVs differ from all other known viruses in ways that challenge traditional views of what viruses are and how they function.

## Polydnaviruses are Beneficial Symbionts Whose Genomes Consist of Two Components

**Figure 1:**
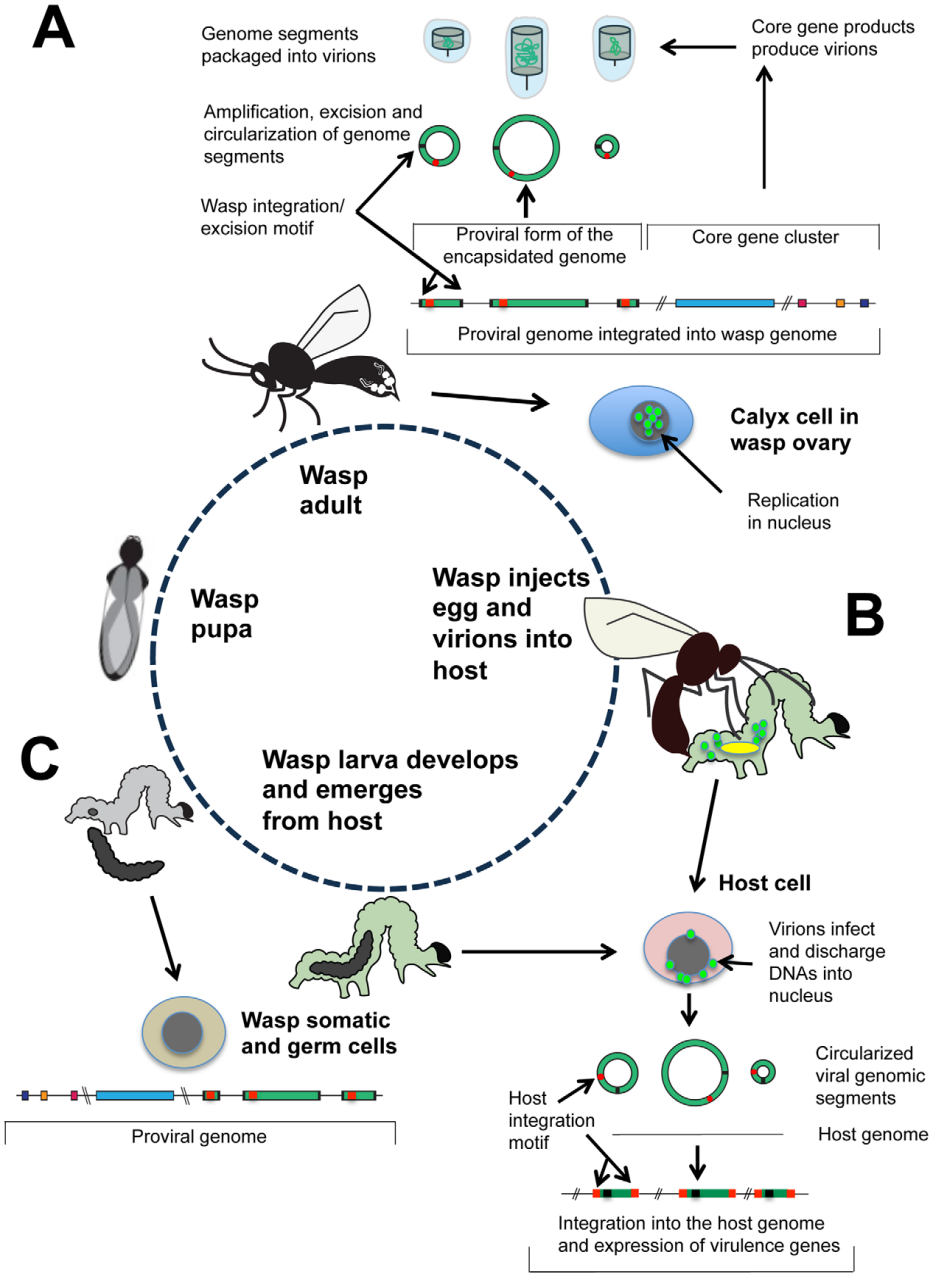
Life cycle and genome organization of BVs. (A) In pupal and adult stage wasps, the proviral genome consists of two components: a domain of core genes (red, yellow, purple) and a domain of tandemly arrayed proviral DNAs (green) that encode virulence genes. The borders of these proviral DNAs are identified by conserved, flanking excision motifs (black). The specific location of BV core genes and proviral DNAs in wasp genomes is currently unknown and is thus indicated by double slash marks. BV replication to produce virions occurs in the nuclei of calyx cells of the female ovary. Coordinate expression of the core genes begins during the wasp pupal stage and continues in the adult stage. This results in the assembly of virions, and the replication, excision, and circularization of proviral DNAs, which are packaged into virions. (B) Wasps inject virions plus one or more eggs containing the proviral genome into the host insect. The egg hatches into a wasp larva that feeds on the host. Virions infect and discharge their DNAs into host cell nuclei, which then rapidly integrate into the genome of the host cell via a second domain present on the viral DNAs named the host integration motif (red). Virulence genes are then transcribed in host cells over the duration required for the wasp larvae to complete their development. The location(s) in the host genome where each viral DNA integrates is currently unknown as indicated by double slash marks. (C) Upon completing development, the wasp larva emerges from the host to pupate, while the host larva dies. Each germ line and somatic cell of the wasp contains the two-component proviral genome.

To appreciate the conceptual challenges PDVs present requires first an understanding of their life cycle. In wasps, PDVs persist in the germ line and somatic cells as an integrated provirus. Replication, in contrast, only occurs in pupal/adult stage females in the nuclei of specialized calyx cells that are located in the ovary (Figure 1A). In the case of bracoviruses (BVs), calyx cells lyse to release single-enveloped virions that accumulate in the lumen of the reproductive tract where eggs are stored (Figure 1A). Wasps then reproduce by injecting eggs containing the proviral genome plus virions into the body cavity (hemocoel) of a host, which is usually the larval stage of a moth (Lepidoptera) (Figure 1B). Virions rapidly infect and discharge their DNAs into the nuclei of host cells, which is followed by the expression of virus-encoded genes. These products have two main functions: 1) they immunocompromise the host, which prevents it from killing the wasp's offspring, and 2) they alter host metabolism and growth, which promotes wasp development and ultimately causes the host to die [4], [5]. However, PDVs never replicate in the wasp's host. Upon completing their development, wasp larvae pupate (Figure 1C). Pupae then emerge as adults to produce a new generation.

Recent studies show that BV genomes exhibit organizational and functional features unlike any other virus group, while also providing important insights into why the activity of PDVs differs between wasps and the hosts wasps parasitize [6]–[9]. In wasps, the proviral genome consists of two components: 1) the core genes that code for essential replication machinery, and 2) regions of DNA that contain virulence genes that are amplified, excised from the wasp genome, and packaged into virions (Figure 1A). Strikingly, the location of the core genes in the wasp genome differs from the location of the proviral DNAs packaged into virions [6]–[9]. Each proviral DNA packaged into virions also possesses conserved, flanking motifs that identify the site of integration/excision from the wasp genome during DNA replication, whereas the core gene-containing domains lack these motifs [6], [10]–[12]. Thus, core genes are expressed in calyx cells to produce virions, but their transmission is entirely vertical and independent of any amplification or encapsidation. Reciprocally, most of the virulence genes of the proviral genome are not transcribed in wasps, but the domains where they reside are amplified and encapsidated in calyx cells for delivery to hosts [6], [8], [13]. In all other cells of the wasp including the germ line, the proviral genome is inactive (Figure 1C). The final novelty is that the genomic DNAs packaged into virions contain a second conserved domain named the host integration motif, which mediates rapid integration into the genome of host cells followed by the continued expression of virulence genes until the wasp's offspring complete their development (Figure 1B) [12]. Overall then, BVs have evolved a life cycle and organizational features that reflect their evolution into beneficial symbionts, which fully depend on wasps for transmission. Reciprocally, wasps fully depend on BV virions for delivery of virulence genes to hosts.

## The Association of BVs with Wasps Is Ancient

**Figure 2:**
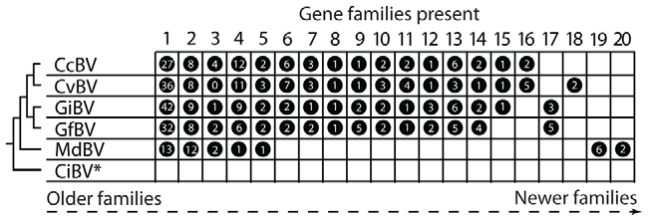
Virulence gene families in the encapsidated genomes of BVs. Each BV isolate is indicated to the left in relation to phylogenetic placement of its associated wasp. The isolates are: *Cotesia congregata* bracovirus (CcBV), *C. vestalis* bracovirus (CvBV), *Glyptapanteles indiensis* bracovirus (GiBV), *G. flavicoxis* bracovirus (GfBV), *Microplitis demolitor* bracovirus (MdBV), and *Chelonus inanitus* bracovirus (CiBV). *C. inanitus* resides in the subfamily Cheloninae while the other wasp species shown are in the subfamily Microgastrinae. The 20 gene families identified from BVs are listed above the figure. Family names derive from predicted function or the presence of a distinguishing motif: 1) protein tyrosine phosphatases; 2) ankyrin-repeat; 3) cysteine-rich; 4) BEN domain; 5) BV4; 6) EP-1 like, homologs of “early expressed protein 1” of CcBV; 7) cystatins; 8) histone-like; 9) C-type lectin; 10) ribonuclease T2; 11) 94K-like, related to baculovirus 94K protein; 12) CrV1-like, homologs of a gene in CrBV; 13) BV2; 14) BV3; 15) Duffy binding-like; 16) BV1; 17) sugar transporter; 18) serine rich; 19) Egf, epidermal growth factor-like; 20) Glc, glycosylated central domain proteins. A circle to the right of an isolate indicates that the gene family is present, while the number inside the circle indicates the number of family members encoded by that isolate. Phylogenetic evidence further suggests families to the left are more ancient, while families to the right have been more recently acquired by particular BV isolates. *The encapsidated genome of CiBV is only partially sequenced. To date, 16 single copy genes are described in the CiBV genome but none of these genes are present in BV isolates outside the genus *Chelonus*.

So when did PDVs evolve? In the case of BVs, all associated wasps reside in a single family named the Braconidae and form a monophyletic assemblage named the microgastroid complex [4]. Fossil calibrations indicate microgastroids evolved 100 million years ago, while survey data indicate the complex consists today of more than 20,000 species that each parasitize only one or a few host species [14], [15]. Consistent with the monophyly of microgastroids and strict vertical transmission, the core gene complement of BV genomes is near fully conserved [6]–[8]. The proviral DNAs BVs package into virions also share organizational features, including that a majority of the virulence genes they encode form multimember families. However, the specific types of virulence genes present differ with isolates from closely related wasps sharing several gene families and isolates from distantly related wasps sharing few or none (Figure 2). Taken together, these data indicate that BVs evolved from the interaction between the common ancestor of microgastroids and a single ancestral virus. In addition, conservation of the core gene component of BV genomes reflects their essential function in virion formation, whereas variation in the virulence genes they encode suggests patterns of acquisition and loss that reflect the diversification of wasps and the hosts they parasitize [9], [16].

## BVs Evolved from a Nudivirus Ancestor

Insights into what BVs evolved from derive from comparing the conserved core genes of their proviral genomes to other viruses. This exercise reveals homologies with the core genes of another group called nudiviruses [6]–[8]. Nudiviruses are relatively poorly studied functionally but are a sister taxon of the Baculoviridae, which is a large family that has been intensively studied as pathogens and expression vectors [17]. The hosts of nudiviruses and baculoviruses are primarily insects, and both groups produce single-enveloped virions that package large circular dsDNA genomes (>100 kbp). Most baculoviruses are virulent pathogens that systemically infect host insects and produce large numbers of virions by cell lysis. Many nudiviruses also establish systemic, lytic infections, but some selectively infect the reproductive organs of hosts and establish latent infections characterized by shut down of most genes expressed during a productive infection and integration into the host genome. BVs thus likely evolved from a nudivirus that established a latent infection in the common ancestor of microgastrine braconids [18]. In contrast to the core genes, BV virulence genes exhibit diverse origins with some being recent acquisitions from wasps themselves, others having been acquired by horizontal gene transfer from other viruses or eukaryotes, and others still being of ancient origin of uncertain history [9], [16].

## How Might the Two-Component Genome of BVs Have Arisen?

The organization of nudivirus genomes and the core genes they encode suggest three features of likely importance in the evolution of BVs into beneficial symbionts. First, nudiviruses package their genomes as a single circular dsDNA into virions, while integrating as a single large linear DNA into the genome of host cells [16], [17]. The two-component genome of BVs, therefore, could have evolved by either duplication of the ancestral genome in the wasp or integration of several copies of the ancestral genome followed by elimination of core genes from some copies and elimination of wasp integration/excision motifs from others [6], [8]. Second, approximately one-third of the nudivirus core genes encode proteins that regulate viral DNA replication, subunits of an RNA polymerase, and other transcription factors. The remaining core genes encode capsid and envelope proteins [17]. The core gene set of BVs includes all of the transcription-related, capsid, and envelope genes present in the nudivirus core gene set. Expression of each BV RNA polymerase subunit also begins at the onset of wasp pupation followed by high-level expression of capsid and envelope genes whose products are confirmed components of BV virions [6]–[8]. In contrast, BV proviral genomes lack homologs of most nudivirus genes thought to regulate viral DNA replication, including a viral DNA polymerase. This suggests replication of the proviral DNAs packaged into virions has shifted from control by viral core genes to control by the DNA replication machinery of the wasp [8]. Third, most baculoviruses and nudiviruses replicate in all cells and stages of infected insects. However, BV replication is stage (pupa), cell (calyx), and sex (female) specific, which suggests BV core genes have acquired features that make them transcriptionally responsive to currently unknown signals produced during the pupal stage of female wasps [9].

## More Bizarre: IchnovirusesAppear to Be Unrelated to BVs

BVs and ichnoviruses (IVs) were assigned to the *Polydnaviridae* because they both package multiple circular dsDNAs into virions and exhibit similar life cycles, albeit the former are associated with braconids and the latter are associated with wasps in another family named the Ichneumonidae [4]. Three lines of evidence, though, indicate that BVs and IVs are not related. First, the braconids that carry BVs are phylogenetically distant from the ichneumonids that carry IVs [4], [15]. Second, BV and IV virions differ morphologically, and while both assemble in the nuclei of calyx cells, BVs are released by cell lysis while IVs are released by budding through the plasma membrane [4]. Third, IV proviral genomes lack homologs of any BV core genes and also encapsidate DNAs that organizationally differ from BVs [4], [19]. Thus, the similar life cycles of BVs and IVs reflect convergent evolution driven by the analogous roles they play in parasitism of hosts by wasps. The viral ancestor(s) of IVs at present is also unknown [19].

## Should PDVs Be Considered Viruses?

Returning to the definition of what a virus is, PDVs clearly replicate inside living cells and produce particles that transfer genes to other cells. Similar to other families of large dsDNA viruses, like the Baculoviridae and Poxviridae, BV genomes also encode a conserved set of replication genes plus other virulence factors from diverse sources that vary among genera and species [16], [20]. Yet, virologists also view viruses as obligate intracellular parasites that use virions to deliver a genome to host cells, which contains all information required to replicate. Strong evidence supports that BVs evolved from an ancestral virus that was just such a parasite. The novelty is that BVs today are obligate beneficial symbionts, which persist entirely from a proviral genome yet produce virions that efficiently deliver genes to other organisms wasps depend upon for survival. Are PDVs still viruses? If we can accept that viruses are not always obligate intracellular parasites, we would suggest the answer is yes.
